# Infectious complications and graft outcome following treatment of acute antibody-mediated rejection after kidney transplantation: A nationwide cohort study

**DOI:** 10.1371/journal.pone.0250829

**Published:** 2021-04-30

**Authors:** Nancy Perrottet, Mario Fernández-Ruiz, Isabelle Binet, Michael Dickenmann, Suzan Dahdal, Karine Hadaya, Thomas Müller, Stefan Schaub, Michael Koller, Samuel Rotman, Solange Moll, Helmut Hopfer, Jean-Pierre Venetz, Vincent Aubert, Léo Bühler, Jurg Steiger, Oriol Manuel, Manuel Pascual, Dela Golshayan, and the Swiss Transplant Cohort Study (STCS)

**Affiliations:** 1 Service of Pharmacy, Lausanne University Hospital (CHUV) and University of Lausanne (UNIL), Lausanne, Switzerland; 2 Transplantation Center, Lausanne University Hospital (CHUV) and University of Lausanne (UNIL), Lausanne, Switzerland; 3 Nephrology and Transplantation Medicine, Cantonal Hospital St. Gallen, St Gallen, Switzerland; 4 Clinic for Transplantation Immunology and Nephrology, University Hospital Basel, Basel, Switzerland; 5 Division of Nephrology, Hypertension and Clinical Pharmacology, Inselspital Bern, Bern, Switzerland; 6 Division of Nephrology and Division of Transplantation, Geneva University Hospitals, Geneva, Switzerland; 7 Division of Nephrology, University Hospital Zurich, Zurich, Switzerland; 8 Basel Institute for Clinical Epidemiology and Biostatistics, University Hospital Basel, Basel, Switzerland; 9 Service of Clinical Pathology, Lausanne University Hospital (CHUV) and University of Lausanne (UNIL), Lausanne, Switzerland; 10 Division of Clinical Pathology, Department of Pathology and Immunology, Geneva University Hospitals, Geneva, Switzerland; 11 Pathology Institute, University Hospital Basel, Basel, Switzerland; 12 Service of Immunology, Lausanne University Hospital (CHUV) and University of Lausanne (UNIL), Lausanne, Switzerland; 13 Visceral and Transplant Surgery, Department of Surgery, Geneva University Hospitals, Geneva, Switzerland; 14 Service of Infectious Diseases, Lausanne University Hospital (CHUV) and University of Lausanne (UNIL), Lausanne, Switzerland; Medical University of Gdansk, POLAND

## Abstract

Acute antibody-mediated rejection (AMR) remains a challenge after kidney transplantation (KT). As there is no clear-cut treatment recommendation, accurate information on current therapeutic strategies in real-life practice is needed. KT recipients from the multicenter Swiss Transplant Cohort Study treated for acute AMR during the first post-transplant year were included retrospectively. We aimed at describing the anti-rejection protocols used routinely, as well as patient and graft outcomes, with focus on infectious complications. Overall, 65/1669 (3.9%) KT recipients were treated for 75 episodes of acute AMR. In addition to corticosteroid boluses, most common therapies included plasmapheresis (56.0%), intravenous immunoglobulins (IVIg) (38.7%), rituximab (25.3%), and antithymocyte globulin (22.7%). At least one infectious complication occurred within 6 months from AMR treatment in 63.6% of patients. Plasmapheresis increased the risk of overall (hazard ratio [HR]: 2.89; *P*-value = 0.002) and opportunistic infection (HR: 5.32; *P*-value = 0.033). IVIg exerted a protective effect for bacterial infection (HR: 0.29; *P*-value = 0.053). The recovery of renal function was complete at 3 months after AMR treatment in 67% of episodes. One-year death-censored graft survival was 90.9%. Four patients (6.2%) died during the first year (two due to severe infection). In this nationwide cohort we found significant heterogeneity in therapeutic approaches for acute AMR. Infectious complications were common, particularly among KT recipients receiving plasmapheresis.

## Introduction

Acute antibody-mediated rejection (AMR) is a potential cause of kidney allograft loss [1–3]. Despite significant advances on the recognition and diagnostic strategies of this clinical entity, the efficacy and safety of the different therapeutic approaches of AMR are not fully established [4]. In 2009, the Kidney Disease Improving Global Outcomes (KDIGO) clinical guidelines recommended a number of treatment alternatives, with or without corticosteroids (grade 2C recommendations), including plasmapheresis, intravenous immunoglobulins (IVIg), anti-CD20 monoclonal antibody (mAb), and lymphocyte-depleting antibodies [5]. These recommendations were recently updated by The Transplantation Society Working Group, and the standard of care for acute active AMR remains plasmapheresis, IVIg (grade 2C) with corticosteroids (expert opinion), and adjunctive therapy in specific settings (grade 2B) [6].

Historically alloantibody removal by plasmapheresis was the first strategy used, associated or not with the immunomodulating effect of IVIg therapy [7–13]. This strategy was then reinforced by aiming to reduce the production of alloantibodies with rituximab, a B-cell-depleting mAb, and bortezomib, a proteasome inhibitor leading to apoptosis of plasma cells [14–19]. More recently, the goal has been to inhibit the complement pathway by using eculizumab, a humanized anti-C5 mAb [20–24]. Nevertheless, none of these strategies has been evaluated in appropriately-powered controlled clinical trials [6, 13]. Beyond efficacy, few studies have systematically assessed the rate of adverse events (and in particular of infectious complications) in patients after AMR treatment. In a recent meta-analysis, only 6 clinical trials of rituximab, bortezomib and eculizumab reported the incidence of infectious diseases complications [13]. Because of a limited number of patients and the heterogeneity in methodology, a detailed epidemiology of infection and its associated risk factors were not reported in these studies.

In view of the lack of high level evidence to guide the optimal choice of acute AMR treatment after kidney transplantation (KT) and the risks associated with the subsequent increase in immunosuppression, we analyzed in a real-life nationwide transplant cohort the current therapeutic practices and the resulting outcomes in terms of efficacy and safety, in particular regarding infectious complications.

## Patients and methods

### Study population

This study was performed within the prospective nationwide Swiss Transplant Cohort Study (STCS, www.stcs.ch, ClinicalTrials.gov: NCT01204944), that was approved by the Ethics Committees of all participating centers (Swissethics BASEC project 2018–022394). Written informed consent was given by all participants, enabling the use of patient- and graft-specific data [25, 26]. This nested study was approved by the Scientific Committee of the STCS, which granted permission to the investigators to use the data from the STCS. For the present study, all kidney transplant recipients recruited in the STCS cohort from May 2008 to May 2014 with a treated acute AMR episode within the first post-transplant year were included. All biopsies were recorded in the STCS database and scored according to Banff classification by each center’s reference pathologist [27–29]. Referent nephrologists at each center were requested to retrospectively confirm the diagnosis of acute AMR and we subsequently analyzed all treated AMR episodes (i.e. requiring the administration of additional anti-rejection therapy). Of note, mixed acute rejection (i.e. AMR together with T-cell-mediated rejection [TCMR]) were also included. In patients diagnosed with >1 episode, each episode was separately analyzed. ABO incompatible transplants were excluded.

### Data collection

The STCS cohort and database have been described previously, and in a recent survey of infectious complications within the STCS [25, 26, 30]. For the present study, a standardized case report form was filled by referent nephrologists to collect additional specific data. Baseline recipient data included age, gender, pre-transplant diabetes, underlying end-stage renal disease, number of transplantations and serostatus for relevant viruses. Transplant-related data included type of donor, donor viral serostatus, complement-dependent cytotoxicity (CDC) crossmatch, number of human leukocyte antigen (HLA) mismatches, type of induction therapy, administration of anti-cytomegalovirus (CMV) and anti-*Pneumocystis* prophylaxis. The following additional data were collected at the time of biopsy-proven rejection: body mass index (BMI), serum creatinine (sCr) and 24-hour proteinuria, presence of DSA, maintenance immunosuppressive and anti-rejection therapies [31]. Follow-up data included sCr levels and 24-hour proteinuria at 3 months after the AMR episode, occurrence of infectious events, one-year patient and graft survival. Induction and maintenance immunosuppressive therapies, as well as prophylaxis regimens, were prescribed as per local guidelines at each center.

### Detection and characterization of donor-specific antibodies

In each center, pre-transplant sera were tested for the presence of anti-HLA antibodies using multiplex technology solid phase assays [31]. Class I (i.e., HLA-A/B) and class II (i.e., HLA-DR/DQ) anti-HLA antibodies were tested and compared with the HLA typing of the donor. DSA were considered positive if mean fluorescence intensity (MFI) values were ≥1,000. Between 2008 and 2011, routine donor HLA typing was done without DQ specificity. Since 2012, all donors were typed for class I and class II HLA molecules using PCR-SSP technology. Detection of DSA against HLA-Cw and HLA-DP was not performed in the present cohort (2008–2014).

### Treatment of AMR episodes

Two different extracorporeal techniques, plasmapheresis or immunoadsorption, were used depending on availability at each center. Immunosuppressive drugs used as anti-rejection therapy included IV methylprednisolone boluses (median total dose [MTD]: 1,500 mg), high-dose IVIg (MTD: 2 g/Kg), rituximab (MTD: 375 mg/m^2^ body surface area), rabbit anti-thymocyte globulin (ATG) (either Thymoglobulin^®^ [MTD: 3 mg/Kg] or ATG-Fresenius^®^ [MTD: 28 mg/Kg]), eculizumab (MTD: 1,800 mg), or bortezomib (MTD: 50 mg/m^2^ body surface area). Of note, the substitutive doses of IVIg (0.4 g/Kg) given after plasmapheresis were not considered as anti-rejection therapy.

### Infectious complications and study outcomes

All infections occurring within the first 6 months after the diagnosis of AMR were retrieved from the STCS database and classified according to the definitions proposed by the Infectious Diseases Study Group of the STCS [25, 26]. For the present study, opportunistic infections were defined as that due to intracellular bacteria (e.g. mycobacteria), herpesviruses (CMV, herpes simplex virus [HSV] and varicella‐zoster virus [VZV]), polyomaviruses (BK virus-associated nephropathy), yeasts (invasive candidiasis and cryptococcosis), molds, *P*. *jirovecii* and parasites (e.g. toxoplasmosis). Only episodes of symptomatic CMV disease were taken into account in the risk factors analysis [32]. Infectious events were classified as proven (confirmed isolated pathogen with clinical signs and/or symptoms and treatment given) or probable (no pathogen identified but in the presence of suspicious clinical signs and/or symptoms leading to a treatment).

Baseline graft function was defined as the mean of the two sCr values prior to the acute increase leading to the diagnosis of AMR. Efficacy of anti-rejection treatment was assessed by comparing graft function at baseline and 3 months after the episode, and categorized as follows: full recovery (±10% of baseline value), no recovery (within ±10% of peak value at AMR diagnosis and/or graft lost), and intermediate recovery (between baseline and peak values). We also analyzed patient and death-censored graft survival at one year after the diagnosis and treatment of AMR.

### Statistical analysis

Quantitative data were shown as the mean ± standard deviation (SD) or median with interquartile ranges (IQR). Qualitative variables were expressed as absolute and relative frequencies. Categorical variables were compared using the χ^2^ test. Student’s t-test or Mann-Whitney U test were applied for continuous variables, as appropriate. Survival and time-to-event curves were plotted by the Kaplan-Meier method and inter-group differences were compared with the log-rank test. Risk factors predicting the occurrence of infection after AMR were assessed by uni- and multivariate Cox regression models. Two different approaches were explored: a “per-patient” analysis, in which only the last AMR episode was taken as reference in patients with >1 episode (i.e. observation period encompassed the entire 6-month interval following the last rejection episode, unless graft loss or death occurred earlier); alternatively, we performed a “per-episode” analysis, in which follow-up was censored at the time of the second/subsequent AMR episode (i.e. observation period could be shorter than 6 months if two consecutive episodes occurred <6 months apart). Associations were expressed as hazard ratios (HRs) with 95% confidence interval (CI). All significance tests were two-tailed. Statistical analysis was performed using SPSS 20.0 (IBM Corp., Armonk, NY) and Prism 6.0 (GraphPad Software Inc., La Jolla, CA).

## Results

### Patient characteristics

Among 1,669 STCS patients who underwent KT between May 2008 and May 2014, we identified 65 recipients (3.9% [95% CI: 3.0–4.8]) with at least one treated episode of acute AMR within the first post-transplant year. Seven patients experienced >1 episode (total of 75 AMR episodes), and one patient received two kidney transplants during the study period (total of 66 KT procedures). Two recipients with combined transplantations were included (kidney-liver and kidney-pancreas). Demographics and clinical characteristics of the study population are summarized in **Table 1**. All patients had a negative pre-transplant T-cell CDC crossmatch, whereas 7.6% (5/66) had a positive pre-transplant B-cell CDC crossmatch. Pre-transplant HLA DSA were detected in 66.7% (44/66) recipients at the time of transplantation. The distribution of the number of DSA (per anti-HLA specificity) in 44 recipients was as follows: 1 DSA in 59.1% (26/44) of recipients, 2 DSA in 18.2% (8/44), 3 DSA in 13.6% (6/44) and ≥4 DSA in 9.1% (4/44). Induction and maintenance immunosuppression as well as prophylaxis regimens are detailed in **Table 2**.

**Table 1:** Demographics and clinical characteristics of the study population (n = 66 kidney transplant procedures performed in 65 patients).

Variable	
Recipient age at transplantation, years [mean ± SD]	46.1 ± 18.5
Gender of recipient (male) [n (%)]	37 (56.1)
Pre-transplant diabetes [n (%)]	9 (13.6)
BMI at the time of rejection, Kg/m^2^ [mean ± SD]	24.6 ± 4.8
BMI at the time of rejection, categorized [n (%)]	
Underweight (<18.5 Kg/m^2^)	5 (7.6)
Normal weight (18.5–25.0 Kg/m^2^)	32 (48.5)
Overweigh (25.0–30.0 Kg/m^2^)	21 (31.8)
Obesity (>30.0 Kg/m^2^)	8 (12.1)
Number of kidney transplant procedures [n (%)]	
First	35 (53.0)
Second	26 (39.4)
Third	5 (7.6)
Underlying end-stage renal disease [n (%)]	
Glomerulonephritis	17 (25.8)
Polycystic kidney disease	14 (21.2)
Diabetic nephropathy	4 (6.1)
Hypertensive nephroangiosclerosis	3 (4.5)
Chronic interstitial nephropathy	3 (4.5)
Reflux nephropathy	3 (4.5)
Congenital nephropathy	2 (3.0)
Unknown	5 (7.6)
Other	15 (22.7)
CMV serostatus [n (%)]	
D+/R+	23 (34.8)
D-/R+	22 (33.3)
D+/R-	11 (16.7)
D-/R-	10 (15.2)
Positive EBV serostatus (anti-EBNA) [n (%)]	65 (98.5)
Positive HBV serostatus (anti-HBc) [n (%)]^a^	4 (6.6)
Positive HCV serostatus [n (%)]	4 (6.1)
Positive HIV serostatus [n (%)]	2 (3.0)
Type of donor [n (%)]	
DBD donor	47 (71.2)
Living related donor	13 (19.7)
Living unrelated donor	5 (7.6)
DCD donor	1 (1.5)
Number of HLA mismatches [median (IQR)]	4 (3–5)
DSA at transplantation [n (%)]^b^	44 (66.7)
Latest PRA value ≥10% [n (%)]^b^	13 (23.2)
Delayed graft function [n (%)]^c^	10 (15.2)

BMI: body mass index; CMV: cytomegalovirus; D: donor; DBD: donation after brain death; DCD: donation after circulatory death; DSA: donor-specific antibody; EBV: Epstein-Barr virus; EBNA: EBV nuclear antigen; HLA: human leukocyte antigen; HBsAg: hepatitis B virus surface antigen; HCV: hepatitis C virus; HIV: human immunodeficiency virus; IQR: interquartile range; PRA: panel-reactive antibody; R: recipient; SD: standard deviation.

^a^ Data on HBV serostatus not available for 5 patients.

^b^ Closest value to transplantation; data on PRA not available for 10 patients.

^c^ Defined as need for dialysis or no decrease in sCr levels in the first week after transplantation.

**Table 2:** Immunosuppression and prophylaxis regimens (n = 66 kidney transplant procedures performed in 65 patients).

Variable	
Induction therapy [n (%)]^a^	
Polyclonal antithymocyte globulin	36 (54.5)
Basiliximab	30 (45.5)
Rituximab	5 (7.6)
Plasmapheresis	3 (4.5)
Eculizumab	1 (1.5)
None	1 (1.5)
Maintenance immunosuppression [n (%)]^b^	
Tacrolimus	54 (81.1)
Serum trough level, ng/mL [mean ± SD]	8.8 ± 4.3
Cyclosporine	11 (16.7)
Serum trough level, ng/mL [mean ± SD]	202.7 ± 95.8
Prednisone/methylprednisolone	65 (98.5)
Prednisone dose, mg/day (median [IQR])	25 (10–40)
Methylprednisolone dose, mg/day (median [IQR])	125 (125–125)
Mycophenolate mofetil	50 (75.8)
Dose, g/day (median [IQR])	2 (1–2)
Enteric-coated mycophenolic acid	12 (18.2)
Dose, mg/day (median [IQR])	1,440 (720–1,440)
Azathioprine	4 (4.5)
Dose, mg/day (median [IQR])	75 (75–75)
Antiviral prophylaxis for CMV [n (%)]^c^	37 (56.1)
Duration of prophylaxis, days [median (IQR)]	120.0 (85–181)
Anti-*Pneumocystis* prophylaxis [n(%)]^d^	65 (98.5)
Duration of prophylaxis, days [median (IQR)]	193 (158–227)

CMV: cytomegalovirus; IQR: interquartile range.

^a^ The sum of percentages exceeds 100% because some patients received more than one induction therapy.

^b^ At the time of rejection.

^c^ Defined as the initiation of IV ganciclovir or oral valganciclovir within the first 2 post-transplant weeks.

^d^ Defined as the initiation of trimethoprim‐sulfamethoxazole, atovaquone, dapsone, or pentamidine initiated during the first 3 months post‐transplant and administered for ≥7 days.

### Acute AMR episodes and anti-rejection therapies

**Fig 1:**
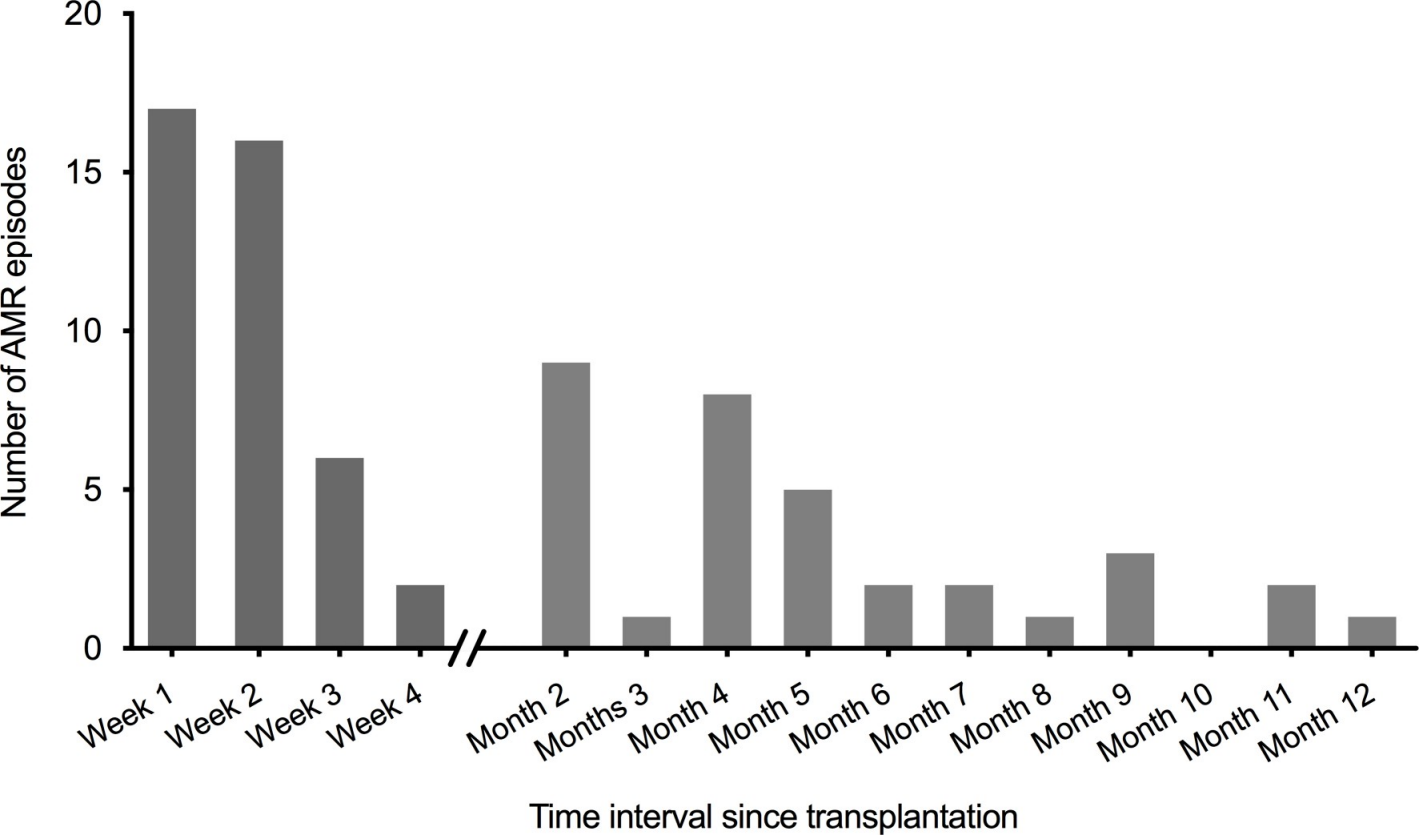
Temporal distribution of episodes of acute AMR in the study cohort. AMR: antibody-mediated rejection.

Most (54.7% [41/75]) acute AMR episodes occurred within the first 4 weeks post-transplantation and 68.0% (51/75) within the first 3 months (**Fig 1**). The median interval from transplantation to the first episode was 14.0 days (IQR: 7.0–93.0). Primary graft non-function was reported in one patient with pre-existing DSA. Of note, of the 75 episodes of acute AMR, 21 biopsies (28%) showed also some signs of T-cell mediated rejection (TCMR). However, all patients had the clinical (graft dysfunction, DSA) and histological features of acute active AMR (glomerulitis, peritubular capillaritis, C4d positivity, and for some also signs of endothelitis).

**Fig 2:**
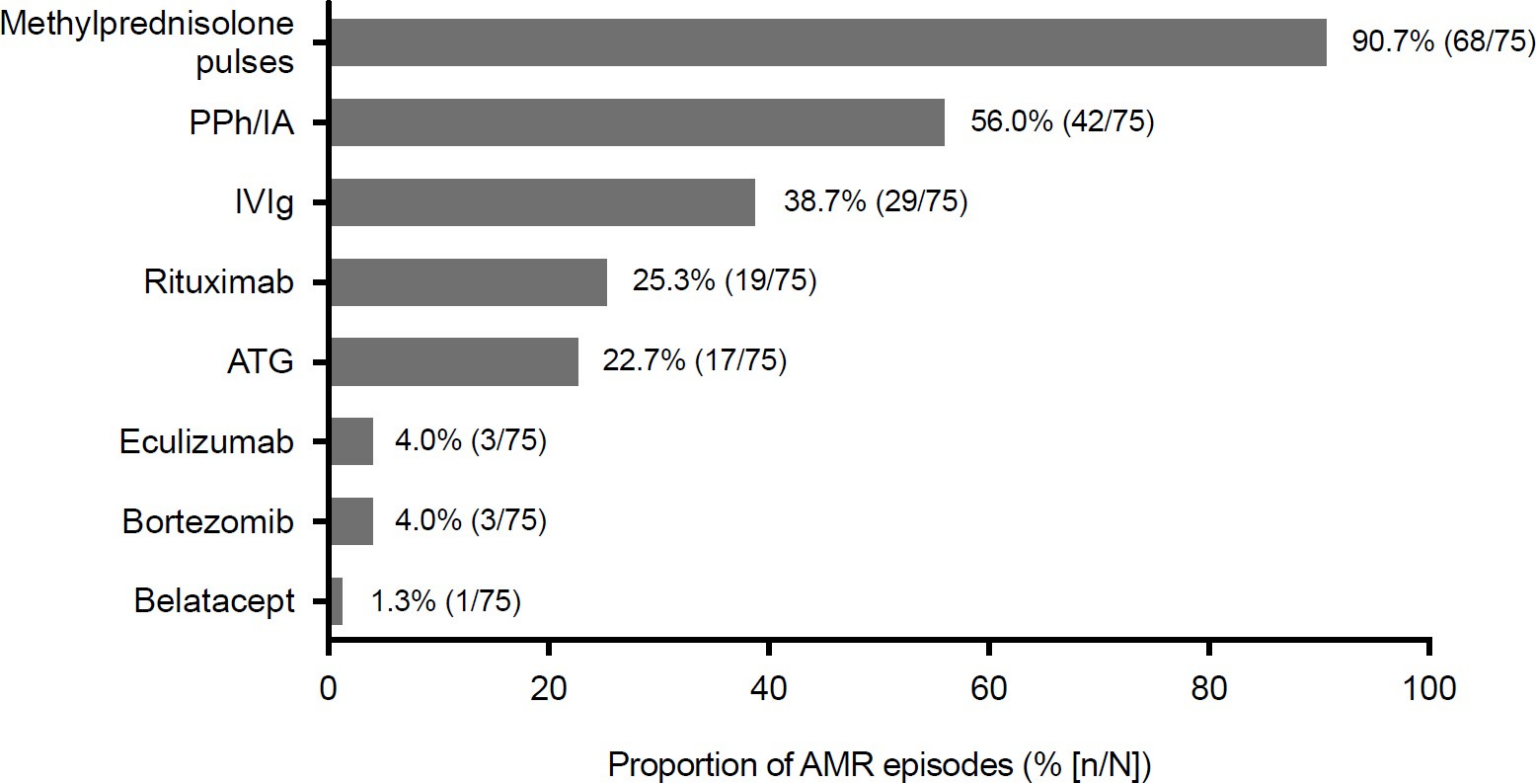
Description of therapeutic approaches in 75 episodes of acute AMR according to individual agents or therapies used. AMR: antibody-mediated rejection; ATG: antithymocyte globulin (different formulations [ATG-Fresenius^®^ or Thymoglobulin^®^]); IA: immunoadsorption; IVIg: intravenous immunoglobulins; PPh: plasmapheresis.

The combination of two different anti-rejection therapies was used in most episodes (44.0% [33/75]), followed by three therapies in 21.3% (16/75), four in 12.0% (9/75) and 5 in 5.3% (4/75). Monotherapy with methylprednisolone boluses was used in 17.3% of cases [13/75]. In addition to methylprednisolone boluses, the most common treatment modalities were plasmapheresis/immunoadsorption (<5 sessions: 45%, with the majority in this group (13/19) having had 5 sessions, 3/19 patients with 4 sessions, 3/19 only 2 sessions; 6–10 sessions: 38%; >10 sessions: 17%) and IVIg, alone or in combination, followed by rituximab and ATG (**Fig 2**). Eculizumab and bortezomib were rarely used. Overall, there were up to 20 different combination regimens. The most common double therapy consisted of plasmapheresis (or immunoadsorption) with methylprednisolone boluses, and the most common triple therapy included plasmapheresis (or immunoadsorption), methylprednisolone and IVIg. One patient with pre-existing DSA underwent splenectomy after multiple sessions of immunoadsorption and plasmapheresis, one dose of rituximab and four doses of bortezomib.

### Infectious complications

Overall, 63.6% (42/66) of recipients developed 96 episodes of infection within the first 6 months following the diagnosis of acute AMR (incidence rate: 0.77 episodes/100 patient-days). The distribution of clinical syndromes and causative agents is detailed in **Table 3**. Most infections were bacterial (42.7% [41/96]). Eighteen episodes of opportunistic infections occurred in 18.2% (12/66) of patients and included BK polyomavirus-associated nephropathy (33.3% [6/18]), CMV disease (27.8% [5/18]), invasive pulmonary aspergillosis (11.1% [2/18]); and *Pneumocystis jirovecii* pneumonia (PCP), *Candida* esophagitis, VZV encephalitis, herpes zoster and mucocutaneous HSV infection (5.5% [1/18], each). Regarding CMV infection, 16.7% [3/18] of episodes occurred in D+/R- recipients (all of them after discontinuation of valganciclovir prophylaxis). The patient who developed VZV encephalitis was treated for severe AMR within the previous month with multiple immunoadsorption and plasmapheresis sessions, methylprednisolone boluses (cumulative dose of 1,250 mg), one dose of rituximab and four doses of bortezomib.

**Table 3:** Clinical syndromes and causative agents of the episodes of infection occurring within the first 6 months after the diagnosis of acute AMR (n = 96).

Clinical syndrome	n (%)
Bacterial infection	41 (42.7)
Urinary tract infection	20
Surgical site infection	2
Respiratory tract infection	4
Gastrointestinal tract infection	3
Skin and skin structures infection	2
Bloodstream infection	10
Urinary source	7
Catheter-related or primary	2
Intra-abdominal source	1
Probable bacterial infection^a^	13 (13.5)
Respiratory tract infection	7
Gastrointestinal tract infection	3
Other	3
CMV infection	18 (18.8)
CMV asymptomatic infection^b^	13
CMV disease	5
Non-CMV viral infection	14 (14.6)
BK polyomavirus-associated nephropathy	6
Biopsy-proven	3
Probable	3
Respiratory tract infection	3
Gastrointestinal tract infection	2
Mucocutaneous HSV infection	1
Cutaneous zoster	1
VZV encephalitis	1
Fungal infection	10 (10.4)
Mucocutaneous candidiasis	4
Intra-abdominal candidiasis	2
Invasive pulmonary aspergillosis	2
*Candida* esophagitis	1
*Pneumocystis jirovecii* pneumonia	1

AMR: antibody-mediated rejection; CMV: cytomegalovirus; HSV: herpes simplex virus; VZV: varicella-zoster virus.

^a^ No pathogen identified.

^b^ Only treated episodes were considered.

**Fig 3:**
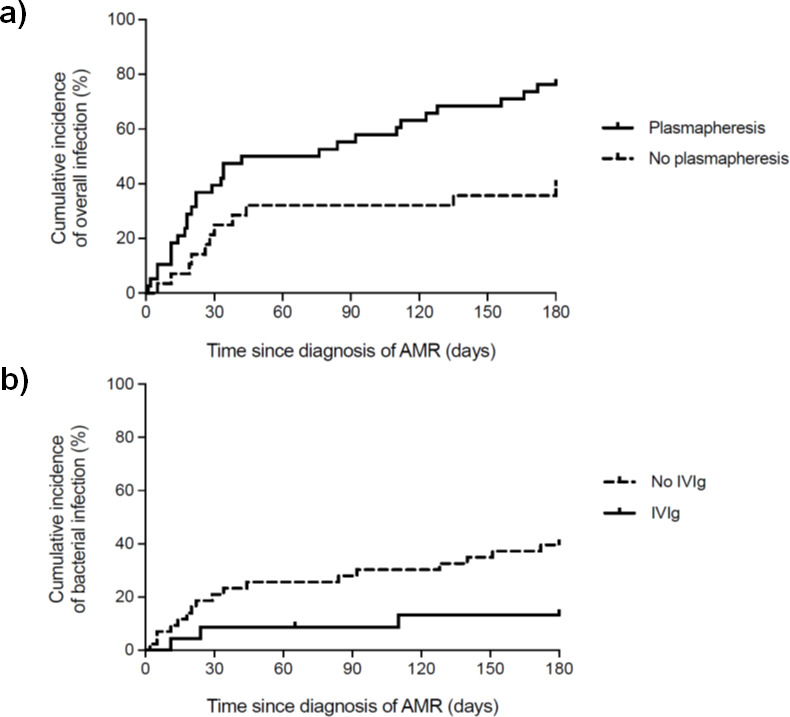
Cumulative incidence of infection within the first 6 months after the diagnosis of acute AMR in the “per-patient” analysis. **(a)** overall infection according to the use of plasmapheresis as anti-rejection therapy (log-rank test *P*-value = 0.002); **(b)** bacterial infection according to the use of IVIg as anti-rejection therapy (log-rank test *P*-value = 0.035). The last episode was taken as reference in patients with more than one rejection episode. AMR: antibody-mediated rejection; IVIg: intravenous immunoglobulins.

**Table 4** shows the results of the multivariate analysis for predicting the occurrence of overall infections in the “per-patient” analysis, in which BMI at the time of rejection (HR [per unitary increment]: 1.10; 95% CI: 1.03–1.17; *P-*value = 0.004) and plasmapheresis (HR: 2.89; 95% CI: 1.46–5.74; *P-*value = 0.002) were risk factors. Accordingly, patients receiving plasmapheresis (either as single therapy or in combination regimens) had higher cumulative incidence of overall infection (**Fig 3A**). These results remain essentially unchanged in the “per-episode” analysis (**S1 Table in S1 File**). When bacterial infection was specifically analyzed, induction with rituximab (HR: 6.57; 95% CI: 2.09–20.71; *P-*value = 0.001) and BMI at the time of rejection (HR [per unitary increment]: 1.10; 95% CI: 1.00–1.21; *P-*value = 0.038) were risk factors, whereas there was a protective effect with IVIg anti-rejection therapy (HR: 0.29; 95% CI: 0.08–1.02; *P-*value = 0.053) (**Table 5**). The cumulative incidence of bacterial infection was significantly lower in patients treated with IVIg-containing regimens (**Fig 3B**). Rituximab induction and plasmapheresis remained as risk factors for bacterial infection in the multivariate model based on the “per-episode” analysis. Regarding opportunistic infection, plasmapheresis was the only risk factor found in the “per-patient” analysis (**S2 Table in S1 File**). When the model was constructed on a “per-episode” basis, plasmapheresis (HR: 5.32; 95% CI: 1.15–27.70; *P-*value = 0.033) also was identified as independent risk factor, and the administration of CMV antiviral prophylaxis (HR: 0.25; 95% CI: 0.08–0.81; *P-*value = 0.022) was protective (**Table 6**).

**Table 4:** Univariate and multivariate analyses of risk factors predicting the occurrence of overall infection within the first 6 months after the diagnosis of acute AMR in the “per-patient” analysis (i.e. the last episode was taken as reference in patients with more than one rejection episode).

	Infection (n = 40)	No infection (n = 26)	*P*-value	Univariate analysis			Multivariate analysis		
				HR	95% CI	*P-*value	HR	95% CI	*P-*value
Age at transplantation, years [mean ± SD]	48.4 ± 17.3	42.5 ± 20.1	0.208						
Gender (male) [n (%)]	21 (52.5)	16 (61.5)	0.470						
Pre-transplant diabetes [n (%)]	7 (17.5)	2 (7.7)	0.465						
BMI at the time of rejection, Kg/m^2^ [mean ± SD]	26.0 ± 4.8	22.5 ± 3.9	**0.002**	1.09^a^	1.03–1.16	0.005	1.10^a^	1.03–1.17	0.004
Previous kidney transplantation [n (%)]	19 (47.5)	12 (46.2)	0.915						
Number of HLA mismatches [median (IQR)]	4 (3–5)	4 (4–5)	0.482						
D+/R- CMV serostatus [n (%)]	7 (17.5)	4 (15.4)	1.000						
Living donor [n (%)]	7 (17.5)	11 (42.3)	**0.027**	0.46	0.19–1.00	0.052	-	-	-
Antithymocyte globulin as induction therapy [n (%)]	22 (55.0)	14 (53.8)	0.927						
Rituximab as induction therapy [n (%)]	5 (12.5)	0 (0.0)	0.148						
Antiviral prophylaxis for CMV [n (%)]	21 (52.5)	16 (61.5)	0.470						
Delayed graft function [n (%)]	6 (15.0)	3 (11.5)	1.000						
More than one episode of acute AMR [n (%)]	3 (7.5)	4 (15.4)	0.420						
Methylprednisolone boluses as anti-rejection therapy [n (%)]	33 (82.5)	25 (96.2)	0.134						
Plasmapheresis as anti-rejection therapy [n (%)]	28 (70.0)	8 (30.8)	**0.002**	2.76	1.39–5.46	0.003	2.89	1.46–5.74	0.002
Immunoadsorption as anti-rejection therapy [n (%)]	3 (7.5)	3 (11.5)	0.673						
IVIg as anti-rejection therapy [n (%)]	12 (30.0)	11 (42.3)	0.305						
Antithymocyte globulin as anti-rejection therapy [n (%)]	9 (22.5)	6 (23.1)	0.956						
Rituximab as anti-rejection therapy [n (%)]	10 (25.0)	8 (30.8)	0.607						
Eculizumab as anti-rejection therapy [n (%)]	2 (5.0)	1 (3.8)	1.000						
Bortezomib as anti-rejection therapy [n (%)]	1 (2.5)	1 (3.8)	1.000						

AMR: antibody-mediated rejection; BMI: body mass index; CI: confidence interval; CMV: cytomegalovirus; HLA: human leukocyte antigen; HR: hazard ratio; IVIg: intravenous immunoglobulins.

^a^ Hazard ratio per unitary increment.

**Table 5:** Univariate and multivariate analyses of risk factors predicting the occurrence of bacterial infection within the first 6 months after the diagnosis of acute AMR in the “per-patient” analysis (i.e. the last episode was taken as reference in patients with more than one rejection episode).

	Bacterial infection (n = 20)	No bacterial infection (n = 46)	*P*-value	Univariate analysis			Multivariate analysis		
				HR	95% CI	*P-*value	HR	95% CI	*P-*value
Age at transplantation, years [mean ± SD]	47.5 ± 17.7	45.5 ± 18.9	0.683						
Gender (male) [n (%)]	8 (40.0)	29 (63.0)	0.083						
Pre-transplant diabetes [n (%)]	4 (20.0)	5 (10.9)	0.437						
BMI at the time of rejection, Kg/m^2^ [mean ± SD]	26.5 ± 5.2	23.8 ± 4.4	**0.037**	1.11^a^	1.01–1.21	0.024	1.10^a^	1.00–1.21	0.038
Previous kidney transplantation [n (%)]	6 (30.0)	25 (54.3)	0.069						
Number of HLA mismatches [median (IQR)]	4 (3–5)	4 (3.8–5)	0.977						
Living donor [n (%)]	5 (25.0)	13 (28.3)	0.785						
Antithymocyte globulin as induction therapy [n (%)]	10 (50.0)	26 (56.5)	0.625						
Rituximab as induction therapy [n (%)]	4 (20.0)	1 (2.2)	**0.027**	5.40	1.77–16.49	0.003	6.57	2.09–20.71	0.001
Delayed graft function [n (%)]	3 (15.0)	6 (13.0)	1.000						
More than one episode of acute AMR [n (%)]	2 (10.0)	5 (10.9)	1.000						
Methylprednisolone boluses as anti-rejection therapy [n (%)]	16 (80.0)	42 (91.3)	0.232						
Plasmapheresis as anti-rejection therapy [n (%)]	15 (75.0)	21 (45.7)	**0.028**	3.04	1.10–8.39	0.031	-	-	-
Immunoadsorption as anti-rejection therapy [n (%)]	1 (5.0)	5 (10.9)	0.659						
IVIg as anti-rejection therapy [n (%)]	3 (15.0)	20 (43.5)	**0.026**	0.29	0.09–0.99	0.048	0.29	0.08–1.02	0.053
Antithymocyte globulin as anti-rejection therapy [n (%)]	6 (30.0)	9 (19.6)	0.358						
Rituximab as anti-rejection therapy [n (%)]	5 (25.0)	13 (28.3)	0.785						
Eculizumab as anti-rejection therapy [n (%)]	1 (5.0)	2 (4.3)	1.000						
Bortezomib as anti-rejection therapy [n (%)]	0 (0.0)	2 (4.3)	1.000						

AMR: antibody-mediated rejection; BMI: body mass index; CI: confidence interval; CMV: cytomegalovirus; HLA: human leukocyte antigen; HR: hazard ratio; IVIg: intravenous immunoglobulins.

^a^ Hazard ratio per unitary increment.

**Table 6:** Univariate and multivariate analyses of risk factors predicting the occurrence of opportunistic infection within the first 6 months after the diagnosis of acute AMR in the “per-episode” analysis (i.e. follow-up was censored at the time of diagnosis of the second or consecutive episodes in patients with more than one rejection episode).

	OI (n = 13)	No OI (n = 62)	*P*-value	Univariate analysis			Multivariate analysis		
				HR	95% CI	*P-*value	HR	95% CI	*P-*value
Age at the time of rejection, years [mean ± SD]	48.2 ± 18.6	47.5 ± 18.5	0.907						
Gender (male) [n (%)]	6 (46.2)	26 (41.9)	0.780						
Pre-transplant diabetes [n (%)]	2 (15.4)	7 (11.3)	0.650						
BMI at the time of rejection, Kg/m^2^ [mean ± SD]	25.8 ± 4.9	24.2 ± 4.5	0.259						
Previous kidney transplantation [n (%)]	6 (46.2)	30 (48.4)	0.883						
DSA at transplantation [n (%)]^a^	10 (76.9)	41 (73.2)	1.000						
DSA at the time of rejection [n (%)]^b^	12 (100.0)	44 (93.6)	1.000						
D+/R- CMV serostatus [n (%)]	0 (0.0)	13 (21.0)	0.108						
Living donor [n (%)]	0 (0.0)	20 (32.3)	**0.015**	0.192	0.03–1.48	0.113			
Antithymocyte globulin as induction therapy [n (%)]	6 (46.2)	36 (58.1)	0.432						
Rituximab as induction therapy [n (%)]	2 (15.4)	3 (4.8)	0.205						
Antiviral prophylaxis for CMV [n (%)]^c^	4 (30.8)	40 (64.5)	**0.025**	0.28	0.09–0.91	0.034	0.25	0.08–0.81	0.022
Antiviral prophylaxis for CMV at the time of acute AMR [n (%)]	3 (8.8)	10 (24.4)	0.076						
Delayed graft function [n (%)]	1 (7.7)	10 (16.1)	0.677						
Previous episode of acute AMR [n (%)]	2 (15.4)	7 (11.3)	0.650						
Methylprednisolone boluses as anti-rejection therapy [n (%)]	10 (76.9)	55 (88.7)	0.364						
Plasmapheresis as anti-rejection therapy [n (%)]	11 (84.6)	29 (46.8)	**0.013**	5.66	1.25–25.55	0.024	5.32	1.15–27.70	0.033
Immunoadsorption as anti-rejection therapy [n (%)]	1 (7.7)	5 (8.1)	1.000						
IVIg as anti-rejection therapy [n (%)]	5 (38.5)	24 (38.7)	0.987						
Antithymocyte globulin as anti-rejection therapy [n (%)]	2 (15.4)	15 (24.2)	0.720						
Rituximab as anti-rejection therapy [n (%)]	4 (30.8)	15 (24.2)	0.727						
Eculizumab as anti-rejection therapy [n (%)]	0 (0.0)	3 (4.8)	1.000						
Bortezomib as anti-rejection therapy [n (%)]	2 (15.4)	1 (1.6)	0.076	7.64	1.68–34.81	0.009	4.80	0.99–23.26	0.051

AMR: antibody-mediated rejection; BMI: body mass index; CI: confidence interval; CMV: cytomegalovirus; DSA: donor-specific antibody; HR: hazard ratio; IVIg: intravenous immunoglobulins; OI: opportunistic infection.

^a^ Data on pre-transplant DSA not available for 6 patients.

^b^ Data on DSA at the time of rejection not available for 16 patients.

^c^ Defined as the initiation of ganciclovir or valganciclovir within the first 2 weeks after transplantation.

### Allograft and patient outcomes

At month 3 after AMR, full recovery of renal graft function was achieved in 66.7% (50/75) of episodes, intermediate recovery occurred in 18.7% (14/75), whereas no recovery was observed in 14.7% (11/75).

**Fig 4:**
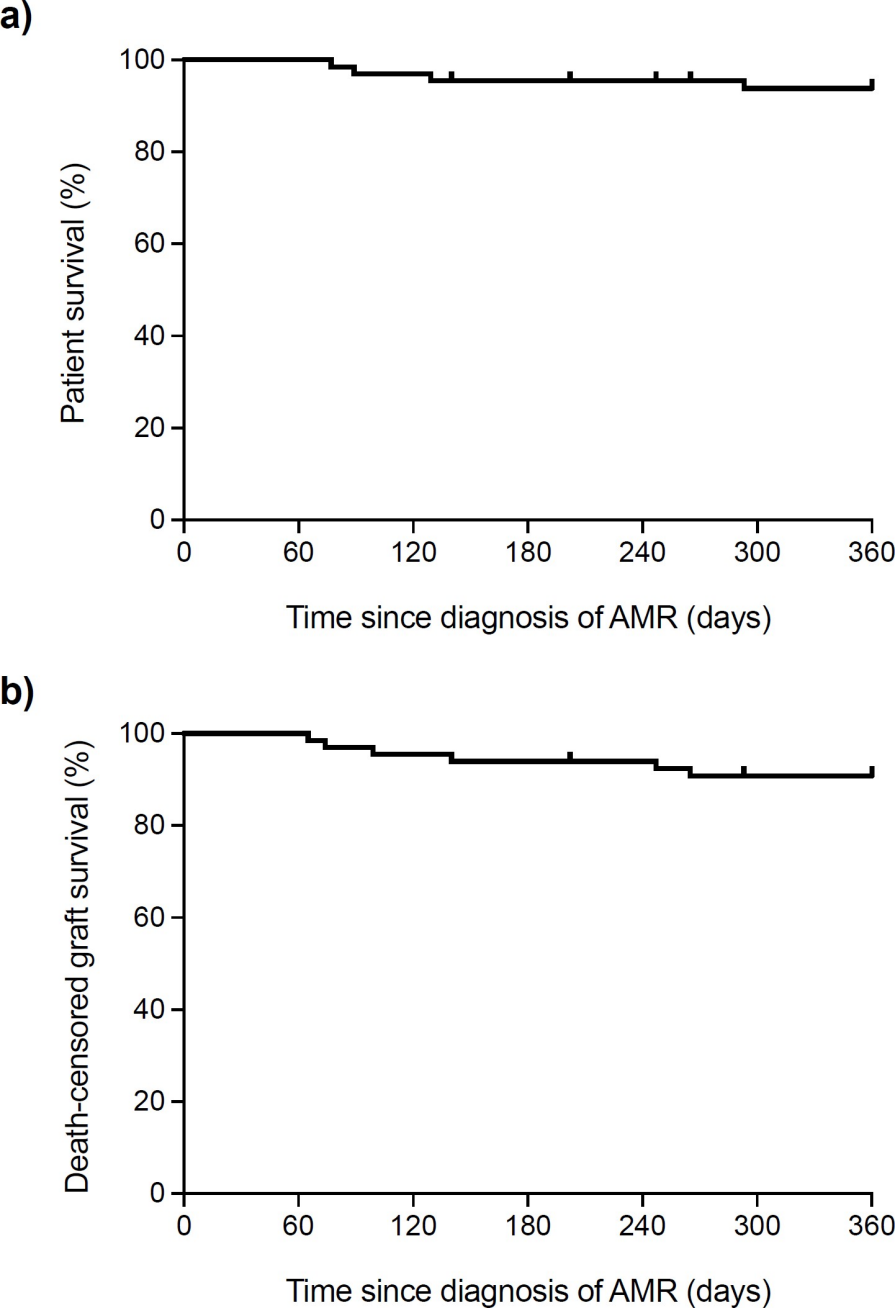
Patient (a) and death-censored graft survival (b) within the first year after the diagnosis of acute AMR in the “per-patient” analysis. The last episode was taken as reference in patients with more than one rejection episode. AMR: antibody-mediated rejection.

At one year after acute AMR, patient survival was 93.8% (**Fig 4A**). Two patients died due to severe infectious complications. The first patient received corticosteroids, multiple plasmapheresis sessions, IVIg and one dose of rituximab following the diagnosis of AMR at day 7 post-transplantation. He successively experienced CMV disease (at day 17) and norovirus infection (at day 49, which led to severe acute tubular necrosis and graft loss). The patient died at month 3 due to multiple organ failure in the context of PCP (despite trimethoprim-sulfamethoxazole prophylaxis). The second patient experienced three different episodes of AMR (at days 7, 35 and 102). Anti-rejection therapies included methylprednisolone boluses, multiple plasmapheresis sessions, ATG, IVIg and two doses of rituximab. After developing different complications (including *Clostridioides difficile* infection, *Klebsiella pneumoniae* bacteremia and BK virus-associated nephropathy), he died due to invasive pulmonary aspergillosis 6 months after transplantation. The causes of death for the other 2 patients were acute coronary syndrome (at month 5 post-transplantation), and gastric cancer (diagnosed at month 6, presumably pre-existing at the time of transplantation).

Death-censored graft survival was 89.4% (**Fig 4B**) at one year after the treatment of acute AMR. The causes of graft loss were chronic AMR (4 cases), primary graft non-function, acute tubular necrosis in the setting of norovirus infection, and hemorrhagic shock due to gastrointestinal bleeding secondary to duodenal ulcers.

## Discussion

In this large nationwide cohort of KT recipients, 3.9% of patients experienced an acute AMR episode that was managed using a large variety of therapeutic approaches within the first year post-transplantation. As the pathogenesis and diagnostic criteria of acute AMR have been refined over the past fifteen years, we did not expect such heterogeneity in the therapeutic practices in this prospective contemporary cohort. Our observation however reflects the lack of validated recommendations regarding the treatment of AMR after kidney transplantation. Despite satisfactory overall patient and allograft outcomes, infectious complications (including episodes of severe and life-threatening opportunistic infections) were however common, particularly bacterial infections (**Table 3**). We found a differential impact of the anti-rejection strategies used on the associated risk of infection, giving support for the individualization of prophylaxis and monitoring regimens in this challenging KT population with acute AMR.

In this cohort, almost two thirds of the patients developed infectious complications within 6 months following acute AMR. We could compare these data to a recent study by Van Delden et al. that investigated the epidemiology of infectious events within the first year after transplantation in the whole STCS database during the same study period between 2008 to 2014 [26]. Regarding the STCS overall kidney cohort, up to 53% of recipients (vs. 63.6% in our subcohort treated for acute AMR) developed at least one episode of infection at 1-year post transplantation. Overall, in a total of 1612 KT recipients 852 patients developed 1964 episodes of clinically relevant infections during the first year after transplantation, among which 66% were bacterial (1299/1964), 28% viral (551/1964), 5% fungal (104/1964) and 0.6% (10/1964) parasitic infections. *E*.*coli* and *Enterococcus sp* were the most common bacterial pathogens with 616/1964 (31.3%) and 217/1964 (11%) of all episodes of infection, respectively, mainly responsible for urinary tract infections. Among viral pathogens, HSV and CMV accounted for 130/1964 (6.6%) and 125/1964 (6.4%) of all episodes of infection, respectively. Infection due to *Candida albicans* was the most common fungal infectious complication in all KT recipients (42/1964, 2.1%). In comparison, while the majority of infections were also due to bacterial pathogens (41/96 episodes, 42.7%) in our patients that were treated for acute AMR, we observed more episodes of opportunistic infections (18.2%), in particular CMV infection, BK polyomavirus-associated nephropathy and invasive pulmonary aspergillosis.

CMV infection represented 18.8% of all episodes, including 5.2% of CMV disease. Of note, a recent case-control study showed that AMR and/or therapy for AMR appears to be a risk factor for CMV infection [33], so that individualized antiviral strategies (i.e. antiviral prophylaxis or rigorously performed preemptive therapy) are important and should be applied in these patients. Most of our patients (56%) received anti-CMV prophylaxis, and the remaining had close monitoring for CMV DNAemia, which may explain the relatively low incidence of CMV disease observed. Besides CMV infection, our data highlight the risk of other severe opportunistic infections associated with the treatment of AMR, such as PCP pneumonia and VZV encephalitis that could be prevented by appropriate prophylaxis. In our study, infection-attributable mortality at one year post-transplantation was up to 3.1% (with both cases developing severe opportunistic infections following multiple anti-rejection therapies), as compared to 0.74% in the whole STCS KT cohort [26].

Despite the large variability in therapeutic modalities used, the data granularity provided by the STCS offered the opportunity to explore some effects of anti-rejection regimens on the susceptibility to infection. We found that the use of plasmapheresis (whatever other therapy it was combined with) significantly increased the risk of overall infection, both in the “per-patient” and “per-episode” analyses. Chung et al. also reported that KT recipients who underwent pre-transplant plasmapheresis associated with rituximab induction had higher cumulative incidence of infection than those without plasmapheresis [34]. It can be hypothesized that plasmapheresis has a deleterious effect on humoral immunity by decreasing serum immunoglobulin levels and complement proteins, thereby impairing opsonophagocytosis and antibody neutralizing activity against pathogens. Accordingly, IVIg administration as anti-rejection therapy (i.e. administered at much higher doses than substitutive doses given after plasmapheresis) exerted a protective effect for bacterial infection. The increased susceptibility to bacterial pathogens among patients receiving induction therapy with rituximab may also support this mechanism, since anti-CD20 agents deplete the B-cell compartment, which can be associated with hypogammaglobulinemia [35]. Infection was also the most common serious adverse event in a recent randomized study involving 38 patients comparing rituximab to placebo for the therapy of AMR (in addition to plasmapheresis, IVIg, and steroids), with opportunistic infections (such as CMV or nocardiosis) also being overrepresented in the rituximab group [17]. Of note, one out of three patients treated with bortezomib was diagnosed with VZV encephalitis, a life-threatening complication. Herpes zoster was the most common infection observed after the introduction of first-generation proteasome inhibitors for hematological malignancies [36] and, therefore, antiviral prophylaxis with acyclovir or valacyclovir is recommended for VZV-seropositive patients with multiple myeloma during induction therapy with bortezomib and for at least 4 weeks after its discontinuation [37]. Such recommendation could also be extended to the KT population, potentially using a drug with large anti-herpes effect such as valganciclovir [38].

In addition to the detailed data on AMR treatment-associated infectious complications, the present study extends previous observations regarding the relatively good prognosis of early acute AMR if diagnosed and treated appropriately and rapidly [12, 39]. The death-censored graft survival observed in our population was close to that recently reported in a larger cohort of KT recipients with AMR and pre-existing DSA [39]. These findings support the possibility of transplanting kidneys into sensitized recipients, using an appropriate induction therapy and a close follow-up strategy aimed at infection prophylaxis and early detection of acute AMR. However, the one-year follow-up of our study may be too short to properly capture medium- and long-term patient and graft outcomes associated with AMR and its treatment. Our data derive from the real-life setting of an ongoing nationwide cohort, emphasizing the broad heterogeneity in single-agent or combination immunosuppressive regimens used to treat acute AMR. Despite lack of strong supporting evidence, a recent systematic review has shown that plasmapheresis and IVIg have become the standard of care for acute AMR due to favorable clinical outcomes reported in most centers [13]. However, the optimal dose of IVIg (median dose of 2 g/Kg in our study) and the number of plasmapheresis sessions (about half of our patients received more than 5 sessions) remain to be better defined, especially in view of the increased infection risk we observed [40].

Our study has several limitations, the most significant being the absence of a control group. Such an ideal control group should have had similar demographics and immunological baseline characteristics, and have developed only acute T-cell mediated rejection (TCMR) without AMR or no acute rejection. Nevertheless, this adjustment was not feasible, since sensitized recipients with DSA pre-transplant tend precisely to develop acute AMR rather than TCMR. This is the reason why we opted to provide some information on the recent study by Van Delden et al., which analyzed the entire KT population within the STCS prospective cohort [26]. On the other hand, our analysis provides new and valuable data on the differential impact of anti-rejection therapies in the setting of acute AMR. Another study limitation is the modest sample size of patients with acute AMR (n = 65). Given the heterogeneity of therapies used, the risk factors for infection identified in the present study would need to be confirmed in larger cohorts. However in this regard our experience can actually be considered as one of the largest series reported to date.

In conclusion, the data obtained from the ongoing nationwide cohort showed a wide heterogeneity in the immunosuppressive therapies used to treat acute AMR after KT. Response to therapy at three months was overall good, with one-year graft and patient survival rates exceeding 90%. However, infectious complications were common after acute AMR treatment. Our findings suggest that such risk varied according to the therapeutic modality, with an increased risk for plasmapheresis while a trend for a protective effect was observed for IVIg on bacterial infection. Finally, our experience emphasizes the need for well-designed prospective controlled studies in AMR among KT recipients in order to define optimal therapeutic regimens in terms of patient and graft outcomes, infectious complications and cost-effectiveness [41].

## Supporting information

S1 File (DOCX)
