# Supplementary material for: Infectious complications and graft outcome following treatment of acute antibody-mediated rejection after kidney transplantation: A nationwide cohort study
Source: PLoS One. 2021 Apr 30;16(4):e0250829. doi: 10.1371/journal.pone.0250829 (PMC8087104; doi:10.1371/journal.pone.0250829)
Supplement: S1 File — (DOCX) [file pone.0250829.s001.docx]

**Perrottet et al., *Infectious complications and graft outcome following treatment of acute antibody-mediated rejection after kidney transplantation: a nationwide cohort study*.**

**S1 File**

**Supplementary Results**

### S1 Table. Risk factors predicting the occurrence of overall infection within the first 6 months after the diagnosis of acute AMR in the “per-episode” analysis (i.e. follow-up was censored at the time of diagnosis of the second or consecutive episodes in patients with more than one rejection episode) [n = 75].

|  | **Infection**  (n = 44) | **No infection**  (n = 31) | ***P*-value** | **Univariate analysis** | | |  | **Multivariate analysis** | | |
| --- | --- | --- | --- | --- | --- | --- | --- | --- | --- | --- |
|  |  |  |  | HR | 95% CI | *P-*value |  | HR | 95% CI | *P-*value |
| Age at the time of rejection, years [mean ± SD] | 50.2 ± 17.3 | 44.1 ± 19.5 | 0.165 |  |  |  |  |  |  |  |
| Gender (male) [n (%)] | 24 (54.5) | 19 (61.3) | 0.561 |  |  |  |  |  |  |  |
| Pre-transplant diabetes [n (%)] | 7 (15.9) | 2 (6.5) | 0.292 |  |  |  |  |  |  |  |
| BMI at the time of rejection, Kg/m^2^ [mean ± SD] | 25.9 ± 4.7 | 22.5 ± 3.6 | **0.001** | ^a^1.09 | 1.03 - 1.17 | 0.003 |  | ^a^1.09 | 1.03 - 1.17 | 0.001 |
| Previous kidney transplantation [n (%)] | 21 (47.7) | 15 (48.4) | 0.955 |  |  |  |  |  |  |  |
| D+/R- CMV serostatus [n (%)] | 8 (18.2) | 5 (16.1) | 0.817 |  |  |  |  |  |  |  |
| Living donor [n (%)] | 7 (15.9) | 13 (41.9) | **0.012** | 0.39 | 0.18 - 0.89 | 0.025 |  | - | - | - |
| Antithymocyte globulin as induction therapy [n (%)] | 23 (52.3) | 19 (61.3) | 0.439 |  |  |  |  |  |  |  |
| Rituximab as induction therapy [n (%)] | 5 (11.4) | 0 (0.0) | 0.073 |  |  |  |  |  |  |  |
| Antiviral prophylaxis for CMV [n (%)] | 24 (54.5) | 20 (64.5) | 0.388 |  |  |  |  |  |  |  |
| Delayed graft function [n (%)] | 6 (13.6) | 5 (16.1) | 0.754 |  |  |  |  |  |  |  |
| Previous episode of acute AMR [n (%)] | 4 (9.1) | 5 (16.1) | 0.475 |  |  |  |  |  |  |  |
| Methylprednisolone boluses as anti-rejection therapy [n (%)] | 36 (81.8) | 29 (93.5) | 0.181 |  |  |  |  |  |  |  |
| Plasmapheresis as anti-rejection therapy [n (%)] | 31 (70.5) | 9 (29.0) | **0.000** | 3.16 | 1.64 - 6.08 | 0.001 |  | 3.03 | 1.57 - 5.84 | 0.005 |
| Immunoadsorption as anti-rejection therapy [n (%)] | 3 (6.8) | 3 (9.7) | 0.687 |  |  |  |  |  |  |  |
| IVIg as anti-rejection therapy [n (%)] | 15 (34.1) | 14 (45.2) | 0.332 |  |  |  |  |  |  |  |
| Antithymocyte globulin as anti-rejection therapy [n (%)] | 10 (22.7) | 7 (22.6) | 0.988 |  |  |  |  |  |  |  |
| Rituximab as anti-rejection therapy [n (%)] | 12 (27.3) | 7 (22.6) | 0.645 |  |  |  |  |  |  |  |
| Eculizumab as anti-rejection therapy [n (%)] | 2 (4.5) | 1 (3.2) | 1.000 |  |  |  |  |  |  |  |
| Bortezomib as anti-rejection therapy [n (%)] | 3 (6.8) | 0 (0.0) | 0.263 |  |  |  |  |  |  |  |
| AMR: antibody-mediated graft rejection; BMI: body mass index; CI: confidence interval; CMV: cytomegalovirus; HR: hazard ratio; IVIg: intravenous immunoglobulins.  ^a^ Hazard ratio per unitary increment. | | | | | | | | | | |

**S2 Table. Risk factors predicting the occurrence of opportunistic infection within the first 6 months after the diagnosis of acute AMR in the “per-patient” analysis (i.e. the last episode was taken as reference in patients with more than one rejection episode) [n = 66].**

|  | **OI**  (n = 12) | **No OI**  (n = 54) | ***P*-value** | **Univariate analysis** | | |  |
| --- | --- | --- | --- | --- | --- | --- | --- |
|  |  |  |  | HR | 95% CI | *P-*value |  |
| Age at transplantation, years [mean ± SD] | 47.3 ± 19.1 | 46.1 ± 18.6 | 0.836 |  |  |  |  |
| Gender (male) [n (%)] | 7 (58.3) | 30 (55.6) | 0.861 |  |  |  |  |
| Pre-transplant diabetes [n (%)] | 2 (16.7) | 7 (13.0) | 0.663 |  |  |  |  |
| BMI at the time of rejection, Kg/m^2^ [mean ± SD] | 26.3 ± 4.8 | 24.2 ± 4.7 | 0.167 |  |  |  |  |
| Previous kidney transplantation [n (%)] | 5 (41.7) | 26 (48.1) | 0.684 |  |  |  |  |
| Number of HLA mismatches [median (IQR)] | 4 (3.3 - 5) | 4 (3 - 5) | 0.646 |  |  |  |  |
| D+/R- CMV serostatus [n (%)] | 0 (0.0) | 11 (20.4) | 0.193 |  |  |  |  |
| Living donor [n (%)] | 0 (0.0) | 18 (33.3) | **0.027** | 0.03 | 0.01 - 5.15 | 0.179 |  |
| Antithymocyte globulin as induction therapy [n (%)] | 6 (50.0) | 30 (55.6) | 0.727 |  |  |  |  |
| Rituximab as induction therapy [n (%)] | 2 (16.7) | 3 (5.6) | 0.221 |  |  |  |  |
| Antiviral prophylaxis for CMV [n (%)]^a^ | 4 (33.3) | 33 (61.1) | 0.111 |  |  |  |  |
| Antiviral prophylaxis for CMV at the time of AMR [n (%)]^b^ | 4 (33.3) | 20 (37.0) | 1.000 |  |  |  |  |
| Delayed graft function [n (%)] | 1 (8.3) | 8 (14.8) | 1.000 |  |  |  |  |
| Previous episode of acute AMR [n (%)] | 2 (16.7) | 5 (9.3) | 0.602 |  |  |  |  |
| Methylprednisolone boluses as anti-rejection therapy [n (%)] | 10 (83.3) | 48 (88.9) | 0.630 |  |  |  |  |
| Plasmapheresis as anti-rejection therapy [n (%)] | 10 (83.3) | 26 (48.1) | **0.027** | 4.56 | 0.99 - 20.80 | 0.050 |  |
| Immunoadsorption as anti-rejection therapy [n (%)] | 1 (8.3) | 5 (9.3) | 1.000 |  |  |  |  |
| IVIg as anti-rejection therapy [n (%)] | 4 (33.3) | 19 (35.2) | 1.000 |  |  |  |  |
| Antithymocyte globulin as anti-rejection therapy [n (%)] | 2 (16.7) | 13 (24.1) | 0.719 |  |  |  |  |
| Rituximab as anti-rejection therapy [n (%)] | 3 (25.0) | 15 (27.8) | 1.000 |  |  |  |  |
| Eculizumab as anti-rejection therapy [n (%)] | 0 (0.0) | 3 (5.6) | 1.000 |  |  |  |  |
| Bortezomib as anti-rejection therapy [n (%)] | 0 (0.0) | 2 (3.7) | 1.000 |  |  |  |  |
